# Investigating the basis of sexual dysfunction during late-onset hypogonadism

**DOI:** 10.12688/f1000research.16561.1

**Published:** 2019-03-25

**Authors:** Tharu Tharakan, Saiful Miah, Channa N. Jayasena, Suks Minhas

**Affiliations:** 1 Department of Urology, Charing Cross Hospital, Imperial College Healthcare NHS Trust, London, UK; 2 Section of Investigative Medicine, Department of Medicine, Imperial College London, London, UK

**Keywords:** Testosterone replacement therapy, late onset hypogonadism, androgen deprivation, andropause, Prostate cancer

## Abstract

Late-onset hypogonadism (LOH) is the term used to describe the decline in serum testosterone levels associated with increasing age in men above 40 years. A number of symptoms are attributed to LOH, but the most common association is that of sexual dysfunction. LOH has recently come under greater scrutiny with the widespread use of testosterone therapy, and concerns regarding the efficacy and safety of testosterone replacement therapy have been raised. In particular, the cardiovascular safety and the beneficial effects of testosterone replacement therapy on general health have been questioned. This review will give an overview of the current evidence for the relationship of LOH and male sexual dysfunction.

## Introduction

A number of studies ^1– 4^ have demonstrated that with increasing age there is an associated decline in serum testosterone levels. Whether this is part of the normal physiological ageing process or a separate pathological entity is controversial. The terminology late-onset hypogonadism (LOH) has been used to describe those patients with an age-related decline in serum testosterone levels, and the most prevalent symptoms are those of sexual dysfunction, particularly decreased morning erections, reduced sexual thoughts, and erectile dysfunction (ED) ^1, 5^.

Interest in LOH has been reported since the mid-20th century. Heller and Myers described the “male climateric” but noted that it was infrequent and a pathologic accompaniment of the ageing process ^6^. Additional terms such as andropause, viropause, partial androgen deficiency of the ageing man, and testosterone deficiency syndrome have all been used to describe this age-dependent testosterone decline. There has been a renewed interest in hypogonadism given improvements in testosterone replacement preparations, the rise in life expectancy, and a recent vogue for male rejuvenation treatments. Moreover, there is evidence ^7, 8^ that testosterone deficiency is associated with metabolic syndrome and potentially a long-term increase in cardiovascular mortality. Therefore, it has been suggested that treatment can encourage weight loss and offset the deleterious long-term cardiovascular risks of metabolic syndrome. However, LOH has recently been placed under scrutiny given the widespread use of testosterone replacement therapy (TRT); in 2011, the global sales of testosterone reached an estimated $1.8 billion ^9^. In addition to this, safety concerns related to the possible increased cardiovascular risk associated with the use of TRT have been raised. Indeed, owing to the media and scientific scrutiny of TRT, there has been a surge of petitioning to the US Food and Drug Administration (FDA) to enforce warnings on medication packaging ^10^. This review aims to give an overview regarding the epidemiology, pathophysiology, diagnosis and management of LOH and also address the controversies associated with this disorder.

## Defining the problem, pathophysiology and epidemiology

Several studies have demonstrated an age-related decline in testosterone and these are summarised in Table 1 ^1– 4, 11^.

**Table 1.** Studies demonstrating age-related testosterone decline.

Reference	Study type	Age range, years	Sample size	Testosterone sample	Results
European Male Ageing Study (EMAS) Wu *et al*. ^1^	Cross-sectional, prospective, multicentre	40–79	3220	Single fasting testosterone (before 10 a.m.)	Total testosterone decline = −0.04 nmol/L year ( *P* <0.001)
The Massachusetts Male Aging Study (MMAS) Feldman *et al*. ^2^	Cross-sectional and longitudinal estimates	40–69	1156	Non-fasting blood samples 4 hours after subject awakening	Cross-sectional results: total testosterone decline = 0.8% per year Longitudinal results: total testosterone decline = −1.6%/year
Baltimore Longitudinal Study (BMAS) Harman *et al*. ^4^	Longitudinal study	22–91	890	Blood samples were in the morning between 7 and 9:30 a.m., after an overnight fast	Total testosterone declined from third to ninth decades. The magnitude of the decrease in total testosterone averaged 0.110 nmol/L (3.2 ng/dL) per year.
New Mexico Aging Study Morley *et al*. ^3^	Longitudinal study	61–87	77	Blood samples drawn between 8 and 11 a.m.	Average rate of testosterone decline was 110 ng/dL every 10 years.
Rancho Bernardo Study Barrett-Connor *et al*. ^11^	Cross-sectional study	50–89	856	Blood samples drawn between 7:30 and 11 a.m.	Bioavailable testosterone and estradiol decreased significantly with age ( *P* <0.01).

## Classification of hypogonadism

Hypogonadism has traditionally been classified into primary or secondary depending upon the aetiology. Primary hypogonadism is due to an intrinsic testicular abnormality resulting in reduced testosterone and elevated luteinising hormone, whereas secondary hypogonadism involves pathology of the pituitary or hypothalamus gland, leading to a disturbance in the hypothalamic–pituitary–gonadal axis and subsequent reduced testosterone and reduced or normal luteinising hormone. Tajar *et al*. ^12^ noted that 9.5% of male patients in the European Male Aging Study (EMAS) had normal testosterone but raised luteinising hormone and this “compensated” hypogonadism was significantly associated with reduced physical activity but not sexual symptoms. The authors felt that compensated hypogonadism should be considered its own clinical entity and that it was a forerunner to overt hypogonadism.

The studies in Table 1 have also documented that associated morbidities play a crucial role in the age-dependent decline of testosterone. Hence, the concept of functional in comparison with organic hypogonadism is emerging ^13, 14^. The former is the result of hypothalamus pituitary testis (HPT) axis perturbation due to the presence of associated morbidities. Hence, if the morbidities are adequately treated and removed, functional hypogonadism is potentially reversible ^13, 14^. The latter, also known as “classical hypogonadism”, is the consequence of organic perturbation of the hypothalamic–pituitary–gonadal axis due to genetic problems, surgery, infective or infiltrative disorders ^14^. However, despite this hypothesis, evidence suggests that ageing per se can induce organic modifications of the hypothalamic–pituitary–gonadal axis.

## Organic

A number of studies ^15, 16^ have shown that with increasing age there is a decline in Leydig cell mass as well as decreased sensitivity to luteinising hormone secretion from the pituitary gland, which leads to a diminished testicular testosterone secretory capacity. Moreover, there is altered neuroendocrine regulation of Leydig cells, leading to a lower threshold for suppression of testosterone production. There is also an independent increase in sexual hormone binding globulin (SHBG) binding capacity, leading to a decrease in bioavailable or free testosterone. These factors are collectively proposed to cause an age-dependent decline in testosterone levels. Furthermore, hypothalamic gonadotrophin-releasing hormone (GnRH) secretion may be reduced in older men when compared with other men ^17^.

## Functional

The Massachusetts Male Aging Study (MMAS) data showed for the first time that healthy men had significantly higher median hormone concentrations at most time points than apparently unhealthy men ^18^. Similarly, data from the EMAS demonstrated that chronic disease, high body mass index and large waist circumference significantly reduced total, free and bioavailable testosterone concentrations ^19^. Moreover, the Hypogonadism in Males (HIM) study demonstrated an association between hypogonadism and the conditions of hypertension, hyperlipidaemia, diabetes and obesity ( *P* <0.001) ^20^.

Grossman and Matsumoto ^14^ argue that the management of functional hypogonadism should not be with hormonal replacement therapy but rather through management of the primary disorder such as obesity, diabetes and metabolic syndrome. Accordingly, weight loss, through either a low-calorie diet or bariatric surgery, may improve testosterone levels ^21^.

However, some studies suggest that the use of supplemental testosterone therapy inhibits adipocyte lipoprotein lipase activity, thereby reducing free fatty acids taken for storage and improving overall body composition ^22^. This weight loss also further improves testosterone levels.

## Association with the metabolic syndrome

Metabolic syndrome is a cluster of several different risk factors that promote atherosclerotic disease. The diagnosis of metabolic syndrome requires the presence of at least three of the following five criteria: increased waist circumference, raised triglycerides, reduced high-density lipoprotein C, raised blood pressure and raised fasting glucose ^23^.

A large body of evidence suggests a bidirectional relationship between LOH and metabolic derangements ^8^.

Braga-Basaria *et al*. ^24^ noted that patients undergoing androgen deprivation therapy (ADT) who developed subsequent hypogonadism had a higher prevalence of metabolic syndrome compared with the non-ADT group ( *P* <0.1) and control group ( *P* <0.03). Several pathophysiological mechanisms underpin this relationship, including the observation that testosterone has been demonstrated to stimulate lipolysis and reduce fatty acid storage ^25– 27^.

Moreover, studies ^28, 29^ have shown that TRT can improve insulin sensitivity in hypogonadal men. Accordingly, a number of systematic reviews and meta-analyses have shown that men with higher testosterone levels (range of 449.6–605.2 ng/dL) had a 42% lower risk of developing type 2 diabetes ^30^.

In line with these data, Yassin *et al*. reported that treatment with testosterone undecanoate long term in an elderly cohort of men with LOH and ED was able to improve several metabolic parameters, including waist circumference and body mass index, cholesterol levels (low-density lipoprotein and total cholesterol), fasting blood glucose and HBA1c levels, and blood pressure over 5 years ^31^. However, it should be recognised that long-term and large placebo-controlled trials evaluating the effects of TRT in patients with metabolic syndrome are still lacking ^32^.

## Epidemiology

The lack of a universally accepted definition for LOH makes estimation of its prevalence a major challenge. The HIM study defined LOH purely on biochemical terms and as a morning total serum testosterone of less than 10.4 nmol/L (300 ng/dL). Mulligan *et al*. ^20^ identified that 38.7% of men over 45 years satisfied the aforementioned criteria. In the Baltimore Longitudinal Study on Ageing, it was found that 19% of men over 60 years had low total testosterone or free testosterone index (total testosterone of less than 11.3 nmol/L (325 ng/dL) or the free testosterone index of less than 0.153 nmol/nmol) ^4^. The EMAS reviewed nearly 3000 men aged 40 to 79 years old. It concluded that only three sexual symptoms (low libido, ED and spontaneous erections) were related to low testosterone levels. Consequently, a strict definition for LOH as a total serum testosterone of less than 11.1 nmol/L (320 ng/dL) combined with the presence of three sexual symptoms (low libido, ED and spontaneous erections) is proposed. With this definition, it is estimated that only 2.1% of men aged 40 to 79 years had LOH ^1^. The MMAS also combined clinical symptoms (loss of libido, ED, and mood or sleep disturbances) and biochemical markers (total testosterone of less than 200 ng/dL or free testosterone of less than 8.91 ng/dL) and identified that between 6 and 12.3% of men had symptomatic androgen deficiency ^33^. In that study, Araujo *et al*. ^33^ estimated that the crude incidence of androgen deficiency was 12.3 per 1000 person-years. Similarly, the Boston Area Community Health survey combined symptoms with biochemical markers (total testosterone of less than 300 ng/dL and free testosterone of less than 5 ng/dL) and estimated that the prevalence of LOH was 5.6% of men in their cohort of 1475 men ^34^.

The International Society of Andrology (ISA), the International Society for the Study of the Aging Male (ISSAM) and the European Association of Urology (EAU) attempted to standardise terminology in 2008 and defined LOH as a “clinical and biochemical syndrome associated with advancing age and characterised by typical symptoms and deficiency in serum testosterone levels” ^35^. Within these guidelines, there was no accepted lower limit for normal, but the consensus was that total testosterone levels above 12 nmol/L (350 ng/dL) do not require testosterone replacement but that men with total serum testosterone levels of less than 8 nmol/L (230 ng/dL) will usually benefit from treatment. These “typical” symptoms are not clearly defined but include ED, mood or cognitive disturbance, muscle weakness, osteoporosis, and body hair and skin changes.

The scientific rationale for the aforementioned definition was based on available scientific data on androgen supplementation therapy and, by their own omission, should be regarded as provisional until higher-level evidence becomes available. Interestingly, they do note that “a correlation between low serum testosterone levels in elderly men and ED has still to be conclusively demonstrated” ^35^. For the purpose of this review, we will be focusing only on the impact of LOH on sexual dysfunction.

## Clinical presentation

Sexual dysfunction is a broad term which encompasses several symptoms, including ED, premature or delayed ejaculation, and low libido. Male sexual function is dependent on several factors, including hormone levels, general fitness, sexual desire, psychological elements as well as the current state of the couple’s relationship ^36^.

Many other studies have shown that sexual frequency decreases with age. Given the complex nature of sexual dysfunction, it is difficult to isolate whether this is a physiological, psychological or pathological consequence of ageing. Moreover, if we were to assume it was pathological, several comorbidities (including drugs) could be attributable. A number of studies have demonstrated the association between LOH and male sexual dysfunction ( Table 2) ^37– 42^.

**Table 2.** Summary of the evidence demonstrating the association between late-onset hypogonadism and sexual dysfunction.

Reference	Study type	Results
Boloña *et al*. ^37^	Systematic review and meta-analysis of 17 RCTs	In hypogonadal men Testosterone treatment caused a large effect on libido (pooled effect size 1.31, 95% confidence interval 0.40–2.25).
Isidori *et al*. ^38^	Meta-analysis of 17 randomised placebo- controlled trials	Meta-analysis showed that in men with an average baseline testosterone level below 12 nmol/L, testosterone treatment moderately improved the number of nocturnal erections, sexual thoughts and motivation, number of successful intercourses, scores of erectile function, and overall sexual satisfaction. However, meta-regression analysis showed that the effects of testosterone supplementation on erectile function, but not libido, were inversely related to the mean baseline testosterone concentration.
Jain *et al*. ^43^	Meta-analysis of 16 studies (five RCTs)	In five RCTs, erectile function response to TRT was 65.4% versus 16.7% ( *P* <0.001).
Tsertsvadze *et al*. ^42^	Meta-analysis of 15 RCTs	Inconsistent data. Results for most trials suggested that TRT was no different from placebo.
Corona *et al*. ^39^	Meta-analysis of 41 RCTs	Forty-one studies were included and supplementary testosterone improved both spontaneous and sex-related erections as well as libido in hypogonadal patients.
Corona *et al*. ^40^	Meta-analysis of 14 RCTs	TRT induced a significant improvement of the erectile function score component compared with placebo ( *P* <0.0001).

RCT, randomised controlled trial; TRT, testosterone replacement therapy.

The above studies have several limitations. Boloña *et al*. ^37^ demonstrated that TRT improved the libido of hypogonadal men but had no impact on erectile function or overall sexual satisfaction. Isidori *et al*. ^38^ noted a significant heterogenicity in effect sizes and risk of publication bias.

The meta-analysis by Corona *et al*. ^39^, who reviewed the effects of testosterone treatment on sexual dysfunction, showed that TRT resulted in an improvement of all sexual function domains, including libido and erectile and orgasmic function. However, the effect was observed only when studies enrolling hypogonadal (total testosterone of less than 12 nM) men were considered. In addition, an inverse relationship between testosterone levels at enrolment and TRT outcomes was documented. Moreover, the same study showed that the effects of TRT were lower in the presence of higher associated morbidities. This may be reflective of a more severe ED. Accordingly, a more recent meta-analysis ^40^ produced by the same group, focusing only on those studies using the International Index of Erectile Function as an outcome measure, provided evidence that TRT alone is able to significantly improve milder forms of ED.

These studies are in contrast with a systematic review by Huo *et al*. in 2016 ^41^. This study included 156 randomised controlled trials (RCTs) and noted that testosterone supplementation did not consistently improve sexual function or satisfaction. Moreover, testosterone supplementation was noted to be ineffective in treating ED. However, this was only a systematic review based on the authors’ evaluation without any statistical analysis as support. Tsertsvadze *et al*. ^42^ demonstrated similar findings in a meta-analysis which did not find any significant improvements of TRT on ED, either individually or when supplemented with PDE5 inhibitors. However, only a limited number of RCTs were included in this study.

## Investigations

The diagnosis of LOH relies on a combination of biochemical and clinical features. This is because men with symptoms of sexual dysfunction and LOH often have a normal testosterone and some asymptomatic patients may have reduced testosterone.

## Testosterone measurement

### Parameters

Serum total testosterone is the favoured biochemical marker to diagnose LOH, and the EAU, ISSAM and ISA recommendations are that a total serum testosterone greater than 12 nmol/L does not require substitution. However, there is no universal agreement on the lower limits of normal testosterone measurements. Sansone *et al*. ^44^ argue that levels below 8 nmol/L are widely considered to require treatment but that levels between 8 and 12 nmol/L represent a grey area where the effects of testosterone are dependent on the patient’s sensitivity to androgens. In this grey area, the authors recommend repeating the total testosterone and also calculating the free testosterone by the combination of total testosterone and SHBG ^45^. When hypogonadism is confirmed, prolactin and gonadotropin levels should be measured to exclude hyperprolactinemia and to better characterise the origin of the problem (i.e is it, primary or secondary?).

### Variability in testosterone measurements

Circulating testosterone levels are subject to circadian variation, so blood testing should be performed in the morning. Furthermore, levels of serum testosterone decrease by 25% following glucose ingestion, so fasting samples are most reliable ^46^.

Furthermore, studies have demonstrated that testosterone measurements can vary greatly. Swerdloff ^47^ noted that 18% of patients who had a normal average testosterone level over 24 hours had single or multiple hypogonadal readings. Hence, usually at least two measurements are required to confirm the diagnosis of hypogonadism.

### Variability in measurement

A wide variety of immunoassays are used to measure serum testosterone. Wang *et al*. ^48^ demonstrated variations in the levels of serum total testosterone dependent on the assay used and when compared to the gold standard of mass spectrometry. However, data from the EMAS showed that results derived from reliable immunoassays are well correlated with mass spectrometry data in men ^49^.

## Assessments of symptoms

The symptom most associated with hypogonadism is low libido. Questionnaires such as the Aging Male Symptom Score (AMS), Androgen Deficiency in Aging Men (ADAM) and the MMAS questionnaire have been shown to have a good sensitivity but lack specificity at diagnosing LOH ^36, 50, 51^. Hence, the use of these self-reported tools for widespread screening of LOH in ageing men should be avoided. In addition, biochemical measurements of testosterone are essential for the diagnosis of LOH. Corona *et al*. validated a 12-item structured interview (ANDROTEST) specifically designed for men seeking consultation for sexual dysfunction, although the sensitivity and specificity of this test at detecting low total testosterone (defined as less than 10.4 n/L) were low: 68% and 65% respectively ^52^.

What complicates the diagnosis of LOH is that sexual dysfunction with ageing is often multifactorial in nature, combining metabolic, cardiovascular and psychological issues. Moreover, there is often a reluctance to seek medical attention for sexual dysfunction, especially in the elderly where there is a belief that sexual dysfunction is a normal process of ageing. Corona *et al*. ^36^ noted that the prevalence of subjects seeking medical care for ED peaked at middle age and decreased thereafter. Therefore, it is difficult to gauge the scale of the issue and also the minority of patients who present for consultation may reflect an extremity.

## Management

### Conservative management

As reported above, obesity is frequently associated with LOH. Camacho *et al*. ^53^ noted that weight loss can reverse the age-related decline in testosterone and free testosterone. Moreover, they demonstrated that, when adjusting forpotential cofounders, a weight decrease of at least 10% led to an increase in testosterone (2.9 nmol/L) and SHBG (13.6 nmol/L) ( *P* <0.01 for both).

Corona *et al*. ^21^ performed a meta-analysis of 24 studies reviewing the impact of weight loss on testosterone levels. In this study, weight loss was associated with a relevant increase in gonadotropins and in bound and unbound testosterone, with a decline in the oestrogen level. The testosterone rise was greater with more weight loss. The above evidence highlights the importance of first trialling lifestyle changes in order to facilitate weight loss and thus avoid the potential side effects related to TRT.

There is an ongoing debate with regard to the impact of improvements in sleep quality and duration on serum testosterone. Wittert ^54^ noted that although some studies ^55, 56^ have shown that treatment of obstructive sleep apnoea (OSA) can improve serum testosterone levels, many others have shown equivocal results. Moreover, when age and obesity are adjusted for, OSA appears to have no direct effect on serum testosterone. Rather than duration of sleep, the timing of sleep seems to be more important on testosterone levels. Schmid *et al*. ^57^ noted that 4.5 hours of sleep restricted to the first half of the night markedly decreased morning testosterone ( *P* ≤0.05).

Studies have identified that the use of PDE5 inhibitors can improve erectile function in hypogonadal patients ^58^ and can increase serum testosterone levels ^59^.

Therefore, Grossmann and Matsumoto ^14^ argue that first-line management should incorporate lifestyle measures as they have the potential to improve testosterone levels and negate the need for TRT. Moreover, this should be accompanied by evidence-based management of the clinical problem which would include treating any ED with a PDE5 inhibitor.

## Infertility

The use of exogenous testosterone causes a negative feedback mechanism in the hypothalamic–pituitary–gonadal pathway resulting in reduced intratesticular testosterone and subsequent impaired spermatogenesis. This mechanism has been demonstrated to be both dose- and duration-dependent ^60^. Our current understanding of the impact of exogenous testosterone on spermatogenesis is from data on trials using testosterone as a male contraceptive. A dose of 200 mg testosterone enanthate weekly by intramuscular injection induced azoospermia at a mean time of 120 days ^61^. Liu *et al*. compared 30 studies evaluating the time of sperm recovery and noted that the typical probabilities of recovery to 20 million per millilitre were 67% (61–72) within 6 months, 90% (85–93) within 12 months, and 100% within 24 months ^62^.

However, other studies have suggested that the adverse effects of exogenous testosterone may be longer-lasting with a proportion of patients not returning to baseline ^63^.

## Human chorionic gonadotropin

Human chorionic gonadotropin (HCG) stimulates Leydig cells to produce testosterone without impairing spermatogenesis. Studies have demonstrated that HCG can improve testosterone and induce spermatogenesis in hypogonadotrophic hypogonadism patients ^64, 65^. Moreover, when low-dose HCG is used in conjunction with exogenous testosterone, it provides a protective effect for intratesticular spermatogenesis and sperm counts are maintained ^66, 67^.

## Selective oestrogen receptor modulators

Selective oestrogen receptor modulators (SERMs) have been shown to stimulate oestrogen receptors and thereby increase the production of gonadotropins and subsequently testosterone. A meta-analysis demonstrated that SERMs were associated with a statistically significant increased pregnancy rate compared with controls (pooled odds ratio [OR] 2.42, 95% confidence interval [CI] 1.47–3.94; *P* = 0.0004) and a significant increase in sperm concentration (weighted mean difference 5.24, 95% CI 2.12–88.37; *P* = 0.001) ^68^.

Clomiphene citrate has been demonstrated to increase testosterone but also improve hypogonadal symptoms ^69, 70^. However, one study indicated that the use of clomiphene citrate is less effective than TRT at increasing serum testosterone levels and improving hypogonadal symptoms and libido ^71^. Enclomiphene citrate is a shorter-acting preparation of clomiphene citrate that has been shown to improve sperm counts but also testosterone levels equivalent to exogenous testosterone ^72^.

## Aromatase inhibitors

Aromatase inhibitors prevent the conversion of estradiol and therefore prevent the negative feedback effect of estradiol on the hypothalamic–pituitary–gonadal axis, thereby increasing testosterone levels ^73^. Leder *et al*. ^74^ demonstrated that the use of anastrozole significantly increased serum testosterone levels but had no significant effect on erectile function compared with control groups. Aromatase inhibitors can commonly cause hot flashes, night sweats, weight gain, insomnia, myalgia and arthralgia. Moreover, patients are at increased risk of osteopenia ^73^.

## Controversies surrounding testosterone replacement therapy

There can be little doubt that sales of testosterone supplementation have increased dramatically in the last decade. Handelsman ^9^ noted that between the years 2000 and 2011, total testosterone sales have increased 12-fold globally, with an estimated rise to $1.8 billion in 2011. Gan *et al*. ^75^ note that, between 2001 and 2010, the number of prescriptions for supplementary testosterone therapy increased by nearly 90% with a 267% escalation of costs to the National Health Service. Of note, a study by the FDA noted that more than 80% of prescription testosterone users in the US were men between 40 and 74 years of age ^76^. This implies that LOH is the disorder contributing to the surge in testosterone sales.

This marked rise in testosterone sales has been aided by the development of more convenient means of administering testosterone. Historically, testosterone supplementation was reliant upon depot injection therapy every few weeks, which caused discomfort and large testosterone level fluctuations, which led to patients complaining of variations in sexual activity and mood. Moreover, these fluctuations could lead to potentially dangerous side effects such as polycythaemia due to the effect of testosterone to stimulate erythropoeisis ^77^. Transdermal testosterone preparations were introduced at the turn of the century and are now the most frequently used. Transdermal testosterone preparations normalise serum testosterone levels with minimal side effects but require active patient compliance. Some patients therefore benefit from taking three monthly depot preparations of testosterone.

Another factor leading to the rise in testosterone sales has been the significant advertising campaigns. Busnelli *et al*. ^78^ note that both direct and indirect marketing campaigns have advertised testosterone replacement as an elixir of eternal youth and social accomplishment and this had promoted disease mongering.

Bandari *et al*. ^79^ performed a systematic review of studies evaluating marketing and testosterone treatment in the US. The authors noted that 10 to 26.6% of men prescribed TRT did not undergo serum testosterone evaluation; of those who did, a significant proportion of men did not meet laboratory criteria for hypogonadism. This illustrates that many patients are being treated with TRT without appropriate investigations, which leads to the potential for drug abuse.

Given that the rise in TRT sales was not evidence-based and given associated safety concerns, the FDA highlighted that prescription of testosterone products are approved only for men with classical hypogonadism, due to disorders of the testicles, pituitary gland or hypothalamus. The same recommendations also emphasise that the benefit and safety of testosterone medications have not been established for the treatment of low testosterone levels due to ageing, even if a man’s symptoms seem related to low testosterone ^80^.

However, it is important to recognise that in animal models of metabolic syndrome obtained by feeding rabbits for 12 weeks with a high-fat diet, metabolic derangements can induce a hypothalamic inflammation leading to an impairment of GnRH secretion. Hence, obesity and its related sequelae can cause organic damage at central levels ^81^.

## Testosterone therapy

Following a trial of conservative management, testosterone replacement can be commenced. The evidence supporting the use of both a PDE5 inhibitor and testosterone replacement to improve ED is equivocal ^82^.

## Safety of testosterone replacement therapy

### Prostate cancer

Huggins and Hodges ^83^ were awarded a Nobel Prize in 1967 for demonstrating that testosterone suppression induced prostate cancer regression. However, historical fears that TRT increased the risk of prostate cancer have been quelled by several studies ^84, 85^. Morgentaler and Traish ^86^ introduced the “saturation model” theory to explain the apparent paradox that ADT can treat prostate cancer but testosterone therapy does not increase susceptibility. This model postulates that whilst testosterone acts as a critical factor to prostatic tissue growth, there is a saturation point for androgen receptors at which further increases in testosterone will have no detrimental effects. Moreover, there is evidence that TRT is safe in patients who have undergone treatment for prostate cancer ^87, 88^ and those undergoing active surveillance ^89^.

### Cardiovascular events

The issue of testosterone therapy and cardiovascular risk is one of controversy and has been investigated by several systematic reviews ^29, 30, 90, 91^. Whereas the FDA offers caution about the potential cardiovascular risks of testosterone therapy, the European Medicines Agency supports the cardiovascular safety of testosterone supplementation, if prescribed and followed in accordance with the current guidelines. Further longitudinal studies are needed to define whether testosterone treatment affects cardiovascular risk in men with LOH.

## Erythrocytosis

Testosterone replacement can cause erythrocytosis ^92, 93^. A meta-analysis incorporating 51 studies demonstrated that exogenous testosterone caused a significant increase in haemoglobin and haematocrit ^90^. However, the pathological mechanism that underpins this haemoconcentration and its potential implications in men is poorly understood ^94^. Moreover, Rhoden and Morgentaler ^95^ note that there have been no reported testosterone-associated thromboembolic events. The evidence linking raised haematocrit and the development of venous thromboembolism is conflicting, and RCTs are needed to evaluate the risk further ^96, 97^.

### Infertility

Testosterone therapy can impair spermatogenesis. This has been discussed earlier; however, if all conservative and alternative therapies have been exhausted, then consideration should be given to nasal testosterone therapy. Masterson *et al*. ^98^ have demonstrated that Natesto increases serum testosterone but maintains gonadotrophin and semen parameters. This is only one study and further research is needed.

## Monitoring

Prior to the start of TRT, prostate-specific antigen (PSA), haematocrit, digital rectal examination and cardiovascular risk assessment should be performed. EAU guidelines ^99^ recommend that treatment be assessed at 3, 6 and 12 months and annually thereafter. Furthermore, the EAU states that there is insufficient evidence to define an optimum serum testosterone level. At these intervals, repeat PSA and haematocrit should be measured; if haematocrit levels increase above 0.54, then dose adjustment or discontinuation is suggested along with phlebotomy. The guidelines do not specify a precise PSA level that mandates investigation but rather that “subjects with a substantial or continuous increase in PSA level need to be investigated to exclude prostate cancer” ^99^. The International Society for Sexual Medicine ^100^ has similar recommendations and monitoring periods but stipulates that a PSA increase of 1.4 ng/nL within 1 year or a PSA velocity of more than 0.4 ng/mL necessitates further investigation.

## Conclusions: Does late-onset hypogonadism result in sexual dysfunction?

LOH remains a diverse clinical entity in terms of diagnosis, investigation and management. There can be no doubt that global testosterone sales have risen substantially in the last century, and it is unclear what proportion of patients have actually benefited from treatment. Moreover, the exact rationale for the use of testosterone in these patients is ambiguous as an FDA study noted that 28% of men who received a new testosterone prescription had no evidence of a prior serum testosterone measurement ^76^. Furthermore, objective evaluation of symptoms with patient-reported outcome measures has inherent difficulties in interpretation with non-validated end points. Sexual dysfunction remains a complex multifactorial condition, and whilst there is evidence to imply an association between LOH and sexual dysfunction, there are no overwhelming data to demonstrate causality between testosterone levels and LOH symptoms in the elderly population. LOH should not be treated purely on biochemical terms. Zitzmann *et al*. ^101^ noted that although the prevalence of loss of libido increases with a total testosterone concentration of less than 15 nmol/L, only 41% of patients with total testosterone below this threshold had loss of libido. Equally, the data that TRT improves sexual function are equivocal. Corona *et al*. ^102^ noted that LOH can be attributed to different sexual symptoms depending on the age group. This highlights the importance of an individualised care plan for each patient presenting with LOH and sexual dysfunction. Given the association of LOH with metabolic syndrome and obesity, all patients should first undergo conservative management via weight loss and optimisation of co-morbidities. Should this fail, it would be appropriate to trial TRT with the caveat that the literature supporting the efficacy of TRT in this clinical setting is inconclusive.
